# Levels of Personality Functioning in Children and Adolescents: A Scoping Review

**DOI:** 10.1192/bjo.2026.11101

**Published:** 2026-06-30

**Authors:** Christopher Mohan, Ayman Osman, Ciaran Clarke

**Affiliations:** 1 College of Psychiatrists of Ireland, Dublin, Ireland; 2 Lucena Clinic, Dublin, Ireland; 3 St Ita’s Intellectual Disability Service, Dublin, Ireland; 4 HSE, Dublin, Ireland; 5 University College Dublin, Dublin, Ireland

## Abstract

**Aims:**

There is increasing recognition of the importance of personality functioning in children and adolescents. The introduction of a dimensional model to conceptualise personality disorders in the ICD–11 and the Alternative Model for Personality Disorders (AMPD) in Section III of the DSM–5, along with the removal of age as a criterion, enables personality disorders to be diagnosed in children and adolescents. This scoping review aimed to explore and map the existing research that included the assessment of the level of personality functioning in this population using an assessment tool.

**Methods:**

This scoping review was pre-registered on Open Science Framework (OSF) Registries and conducted in accordance with JBI methodology for scoping reviews. Five databases were searched: Medline (EBSCO), PsycINFO (APA), CINAHL Complete, Embase, Cochrane Library. A limited search of grey literature from Google Scholar was conducted. Articles identified through citation searching of included sources of evidence were also included. A librarian was consulted to develop the search strategy. Key words and synonyms related to (i) children and adolescents, (ii) levels of personality functioning and (iii) assessment. Sources of evidence published up to 2025 and in any language were included. Screening was completed by two reviewers with conflicts resolved through consensus discussion and a third reviewer. One reviewer completed data extraction, and a second reviewer checked a proportion of the data. AI tools were used to translate articles notpublished in English. Covidence software was used to manage data and facilitate the identification of duplicate articles.

**Results:**

The search strategy retrieved 5191 sources of evidence, of which 544 duplicates were removed, 4501 were excluded during abstract screening, 90 during full-text review, and 2 were merged, resulting in a total of 55 sources of evidence in the final analysis. Publication dates ranged from 2011 to 2025, with 51% of publications since 2023. The majority (n=49) were research studies undertaken in 14 countries. Of which, 34 included a clinical population. Eighteen assessment tools were identified; 16 were self-report tools and were used in the majority (n=40) of research studies. The Levels of Personality Functioning Questionnaire for Adolescents (LoPF-Q 12-18) was the most frequently used measure in the studies (n=22).

**Conclusion:**

There is increasing research on assessing levels of personality functioning in children and adolescents. Included sources of evidence report that future research should include larger, longitudinal evaluations, multi-informant data, and evaluate the utility of assessment tools across different clinical, demographic, geographical, and cultural settings.

